# Assessing use of a printed lifestyle intervention tool by women with borderline gestational diabetes and their achievement of diet and exercise goals: a descriptive study

**DOI:** 10.1186/s12884-016-0825-z

**Published:** 2016-03-01

**Authors:** Shanshan Han, Philippa F. Middleton, Thach S. Tran, Caroline A. Crowther

**Affiliations:** Australian Research Centre for Health of Women and Babies (ARCH), Robinson Research Institute, School of Medicine, Discipline of Obstetrics and Gynaecology, The University of Adelaide, 72 King William Road, North Adelaide, 5006 South Australia Australia; Women’s and Children’s Health Research Institute, 72 King William Road, North Adelaide, 5006 South Australia Australia; Garvan Institute of Medical Research, 384 Victoria Street, Darlinghurst, 2010 NSW Australia; Liggins Institute, The University of Auckland, Private Bag 92019 Victoria Street West, West Auckland, 1142 Auckland New Zealand

**Keywords:** Borderline gestational diabetes mellitus, Pregnant women, Diet, Exercise, Printed lifestyle intervention tool, Descriptive study

## Abstract

**Background:**

The purpose of this study is to assess use of a booklet by pregnant women to record and assist dietary and lifestyle changes; to describe diet and exercise goals set during the initial lifestyle discussions; and to assess achievement of goals.

**Methods:**

Participants were women with borderline gestational diabetes who received a printed pregnancy record booklet, as part of a randomised trial, to record and set monthly goals for diet and exercise. Outcomes included women’s use of the booklets and their achievement of dietary and exercise goals after 1 month.

**Results:**

Fifty-six women returned their used pregnancy record booklets and were included in this study. These women set a total of 197 dietary goals and 65 exercise goals. In the first month, over 80 % of dietary goals that targeted grains, dairy and overall diet were achieved, but only 20–30 % of goals about vegetables, and foods high in fat, sugar and/or salt were achieved. After 1 month, women had achieved 86.4 % of their exercise goals to maintain their current level of activity, but only 25.0 % exercise goals to increase walking during pregnancy.

**Conclusions:**

Women who used pregnancy record booklets reported good achievement rates for goals related to grains, fruits, dairy and overall diet, but they were less likely to be successful in achieving goals to increase intake of vegetables, and limit foods that high in fat, sugar and/or salt. Maintaining an active lifestyle during pregnancy was feasible for women although increases in physical activity were less often achieved.

Using a pregnancy record booklet may be helpful in assisting and encouraging behavioural changes, although further investigations of long-term effects and in different populations are warranted.

**Electronic supplementary material:**

The online version of this article (doi:10.1186/s12884-016-0825-z) contains supplementary material, which is available to authorized users.

## Background

Gestational diabetes mellitus (GDM) is defined as hyperglycaemia first recognised during pregnancy [1, 2]. Due to the increasing rates of maternal obesity and type two diabetes mellitus and the trend towards older maternal age, the prevalence of GDM is increasing worldwide [3–6].

GDM is associated with a range of adverse health outcomes for both the mothers and their babies [7–10]. Behavioural management, involving dietary and exercise interventions, has been found to be beneficial for women with pregnancy hyperglycaemia [11–15]. Medical nutrition therapy (MNT) has been recommended as the primary therapeutic strategy for managing pregnancy hyperglycaemia [16–19].

Printed lifestyle intervention tools have been frequently used for providing interventions and facilitating behavioural modifications in women with GDM [20–22]. However, there are few reports about the usefulness of these strategies for women with pregnancy hyperglycaemia.

This descriptive study was nested within a multicentre, randomised controlled trial that commenced in 2008, investigating the effect of dietary and lifestyle advice for women with borderline gestational diabetes [defined as a positive 50 g oral glucose challenge test (OGCT) (1 h venous plasma glucose ≥7.8 mmol/L) followed by a normal 75 g oral glucose tolerance test (OGTT) (fasting venous plasma glucose <5.5 mmol/L and a 2 h glucose <7.8 mmol/L)] (the IDEAL Study) [23]. As outlined in the published study protocol, women between 24^0^ and 34^6^ weeks’ gestation with a singleton pregnancy, classified as having borderline GDM were eligible for the IDEAL Study [23]. Women were randomised into either the ‘Intervention Group’ or the ‘Routine Care Group’ [23]. Those in the Intervention Group had a lifestyle discussion with a dietitian as soon as they enrolled in the study and were given the pregnancy record booklets as part of the study intervention [23]. Women in the Routine Care Group received standard antenatal care with no lifestyle discussion or pregnancy record booklet provided [23].

The present study aimed to assess women’s use of pregnancy record booklets provided to assist their dietary and lifestyle changes; to describe the woman’s diet and exercise goals set during the initial lifestyle discussion; and to describe goals achieved after 1 month.

## Methods

### Participants and procedure

Women in the IDEAL study Intervention Group who received pregnancy record booklets during their initial lifestyle discussions with a dietitian, were invited to return their pregnancy record booklet after their babies were born. To facilitate booklet return via post, a reply paid envelope was attached to the pregnancy record booklet and women were made aware of it at the initial lifestyle discussion. Women were advised to bring their pregnancy record booklet when attending antenatal appointments and when admitted for childbirth. Women who returned and used their booklets at least once were included in this study.

### The pregnancy record booklet

The pregnancy record booklet was designed for women to record their existing diet and exercise patterns and to set diet and exercise goals for the coming month (see Additional file 1 for sample pages), based on published recommendations and the best available evidence at that time [24, 25]. During the initial lifestyle discussion, the woman’s diet and exercise were reviewed and diet and exercise goals aiming for adequate nutrient intake and optimal glycaemic control were set for the next month. Factors including the woman’s age, pre-pregnancy weight, activity level, current dietary intake and weight gain for the current and any previous pregnancies were considered when advising women about their diet and exercise goals [23].

Individualised dietary and lifestyle goals focused on the quantity and quality of food consumption. Diet quantity was prespecified in the pregnancy record booklet as daily intakes of food from the categories of bread/cereal (grains), vegetable, fruit, dairy, meat and protein-rich food and foods high in fat, sugar and/or salt. Diet quality included choosing low glycaemic index foods, low fat dairy, higher fibre food varieties, lean meat, and using unsaturated oils and limiting fats and oils. Women’s exercise goals focused on the type, duration, frequency and intensity of the physical activity, which were tabulated in the pregnancy record booklet.

One month after the initial lifestyle discussion, by using the pregnancy record booklet, women reviewed their dietary and exercise habits, set and documented new goals for the next month. Women were requested to review and reset their diet and exercise goals each month during pregnancy. If women had any concerns and/or questions around their diet and exercise after the initial lifestyle discussion, they were encouraged to talk with their attending midwives or obstetricians.

### Analysis

Information recorded in the woman’s pregnancy booklet was entered into the study database. Dietary data about quantity and quality of food consumed were transcribed, coded and categorized into seven categories as prespecified in the pregnancy record booklet including bread/cereal (grains), vegetable, fruit, dairy, meat and protein rich food, foods high in fat, sugar and/or salt and overall dietary goal. Exercise data were transcribed and coded, and analysed thematically by systematic comparisons based on grounded theory methods [26]. Women’s use of pregnancy record booklet during pregnancy was analysed using SAS software version 9.3 (SAS Institute Inc., Cary, NC, USA).

## Results

### Participants and booklet use

A total of 56 women, who completed their initial lifestyle discussion at a mean (standard deviation) gestation of 31.6 (1.7) weeks, used booklets at least once by the end of pregnancy and were included in the study.

Women who used the booklet compared with all women who received a booklet from the randomised trial, were more likely to be primiparous (66.1 % versus 50.6 %), of Asian ethnicity (21.4 % versus 16.2 %), have a family history of diabetes (51.8 % versus 37.5 %) or hypertension (51.8 % versus 36.8 %); but were less likely to be obese (9.4 % versus 19.9 %), smoke (9.1 % versus 16.9 %) or be socially disadvantaged (12.5 % versus 21.8 %) (Table 1).

**Table 1:** Baseline characteristics: women who used pregnancy record booklet versus all women who received booklet

Characteristics	Women who returned booklets and used at least once (*n* = 56)	Women who received booklets (*n* = 358)
Maternal age (years)^a^	31.3 (4.5)	30.6 (5.1)
Primiparity	37 (66.1)	181 (50.6)
BMI Category		
-Underweight (<18.5 kg/m^2^)	3 (5.7)	8 (2.3)
-Normal (18.5–24.9 kg/m^2^)	30 (56.6)	182 (52.6)
-Overweight (25–29.9 kg/m^2^)	15 (28.3)	87 (25.1)
-Obese (≥30 kg/m^2^)	5 (9.4)	69 (19.9)
Race		
-White	43 (76.8)	265 (74.0)
-Asian	12 (21.4)	58 (16.2)
-Other	1 (1.8)	35 (9.8)
Smoker	5 (9.1)	59 (16.9)
Family history of diabetes	29 (51.8)	133 (37.5)
Family history of hypertension	29 (51.8)	130 (36.8)
Socioeconomic status^b^		
-Most disadvantaged	7 (12.5)	78 (21.8)
-Disadvantaged	10 (17.9)	56 (15.6)
-Average	15 (26.8)	72 (20.1)
-Advantaged	14 (25.0)	83 (23.2)
-Most advantaged	10 (17.9)	69 (19.3)

Figures are number and percentage

*BMI* body mass index

^a^Mean and standard deviation

^b^As measured by the Australian Bureau of Statistics Socio-Economic Indexes for Areas [29]

Use of the booklet by women included review of their current diet, dietary goal setting, their exercise pattern and exercise goal setting. Of the 56 women, all used the booklet at least once for reviewing their diet and 52 (92.9 %) used the booklet for setting their dietary goals for the next month; 53 (94.6 %) women used their booklets at least once for reviewing their exist pattern of exercise and 44 (78.6 %) used the booklet for setting exercise goals for the next month.

### Dietary goal setting and achievement

Dietary goals were set from seven different food categories as prespecified in the pregnancy record booklet, including bread/cereal (grains); vegetable; fruit; dairy; meat and protein rich food; foods high in sugar, fat and/or salt, or not belonging to the categories above; and overall dietary goals which focused on the overall quantity or quality of the diet (Table 2). The most frequently targeted dietary goal was to reduce foods high in sugar, fat and/or salt, followed by overall dietary goals of maintaining good dietary patterns and/or habits.

**Table 2:** Dietary goals women set and whether achieved after 1 month by category

Women’s goal setting and achievement	Set goals	Achieved goals	Improved but did not achieve	Did not change	Worsened	Unknown
Dietary goals	*N* ^a^ (%)	*N* (%^b^)	*N* (%)	*N* (%)	*N* (%)	*N* (%)
Bread, cereal (grains) group						
22 goals set by 20 women	20 (38.5)	17 (85.0)	2 (10.0)	2 (10.0)	-	-
−Increase overall daily intake −Choose low GI food varieties −Choose wholemeal/wholegrain bread −Choose wholemeal flour −Choose wholemeal pasta −Reduce gluten free bread						
Vegetable						
22 goals set by 22 women	22 (42.3)	7 (31.8)	4 (18.2)	6 (27.3)	1 (4.5)	4 (18.2)
−Increase daily vegetable intake −Less potato and sweet potato						
Fruit						
26 goals set by 25 women	25 (48.1)	18 (72.0)	3 (12.0)	2 (8.0)	-	3 (12.0)
-Increase overall daily fruit intake -Reduce overall daily fruit intake -Reduce daily fruit juice intake -Limit tropical fruit						
Dairy						
27 goals set by 25 women	25 (48.1)	20 (80.0)	1 (4.0)	2 (8.0)	-	2 (8.0)
-Choose low fat dairy -Increase dairy intake -Reduce dairy intake						
Meat and protein rich food						
26 goals set by 25 women	25 (48.1)	15 (60.0)	1 (4.0)	5 (20.0)	2 (8.0)	3 (12.0)
-Reduce overall meat intake -Increase overall meat intake -Choose lean meat -Increase red meat intake						
Extra food^c^						
40 goals set by 35 women	35 (67.3)	8 (22.9)	2 (5.7)	4 (11.4)	-	23 (65.7)
-Limit food high in fat, sugar and/or salt -Limit fat and oil -Change the type of fat used in cooking -Replace buttered toast with cereal and milk -Reduce coffee						
Overall dietary goal						
34 goals set by 23 women	23 (44.2)	22 (95.7)	-	-	-	2 (8.7)
-Maintain current dietary intake (quantity) -Maintain current good dietary habits (quality) -Reduce the overall food serving sizes						

*GI* Glycaemic Index

^a^Table subtotals do not add to 100 % because women may have set multiple goals for one goal category

^b^percentage of women who set goals

^c^includes food high in fat, sugar and/or salt, and those do not belong to any of the five main food groups

#### Bread/ cereal (grains) group

The grains group was targeted by the fewest women (*n* = 20, 38.5 %) among the seven goal categories. Of the 22 goals, 11 were set to improve diet quality by choosing low glycaemic index food (GI) such as brown rice and pasta or higher fibre food varieties such as wholemeal bread (Table 2). The remaining goals were set to increase daily intake of food from this group. Of these, only two goals (20.0 %) were set to meet recommendations (Table 3).

**Table 3:** Dietary goals women set in relation to dietary recommendations^a^

Food groups	Met dietary recommendation	More than dietary recommendation	Less than dietary recommendation	Unknown	Total
Bread, cereal	2 (20.0)	0 (0.0)	6 (60.0)	2 (20.0)	10
Vegetable	11 (52.4)	0 (0.0)	7 (33.3)	3 (14.3)	21
Fruit	10 (52.6)	3 (15.8)	3 (15.8)	3 (15.8)	19
Dairy	12 (85.7)	1 (7.1)	0 (0.0)	1 (7.1)	14
Meat	4 (21.1)	10 (52.6)	3 (15.8)	2 (10.5)	19

Figures are number and percentage

1. Bread/cereal group: 4–6 serves/ day

2. Vegetable: 5–6 serves/ day

3. Fruit: 2–4 serves/ day

4. Dairy: 2–3 serves/ day

5. Meat/ other protein: 1.5 serves/ day

^a^According to Australian Guide to Healthy Eating (1998) [24]

After 1 month, the majority of women (*n* = 17, 85.0 %) achieved their goals, which included choosing low GI food or higher fibre food varieties (10 goals) and increasing daily serves according to the dietary recommendation (seven goals). Two (10.0 %) women who set two goals to increase their daily intake of bread and/or cereal (grains), improved their bread and/or cereal (grains) intake after 1 month, but did not reach their goals.

#### Vegetable group

Twenty-two (42.3 %) women set a total of 22 dietary goals relating to vegetables during the initial visit (Table 2). Almost all goals (*n* = 21, 95.5 %) aimed to increase daily vegetable intake (Table 2). Of these, 11 (52.4 %) met dietary recommendations and seven (33.3 %) partially met the recommendation (Table 3).

After 1 month, only seven (31.8 %) women achieved their goals, which included increasing vegetable intake (six goals) and reducing starchy vegetables (one goal). One (4.5 %) woman, who set a goal to increase her vegetable intake, reported decreased vegetable intake after 1 month (Table 2).

#### Fruit group

During the initial lifestyle discussion, 25 (48.1 %) women set 26 goals relating to fruit (Table 2). Most goals (*n* = 19, 73.1 %) were to adjust the serves of daily fruit intake according to the dietary recommendations, including 16 goals to increase fruit intake and three goals to reduce daily fruit intake (Table 2). For optimal glycaemic control, other goals included limiting high GI fruit such as tropical fruits (five goals) and limiting fruit juice intake (two goals) (Table 2). Of the 19 goals to adjust daily serves, about half (*n* = 10, 52.6 %) met the dietary recommendations, three (15.8 %) were more than the recommendations and another three (15.8 %) were less than the dietary recommendations (Table 3).

After 1 month, most (*n* = 18, 72.0 %) women achieved their dietary goals of increased fruit intake (9 goals), reduced tropical fruit (5 goals) and reduced fruit intake (4 goals). Three (12.0 %) women who set three goals of increased fruit intake reported increased intake but did not achieve their goals after 1 month (Table 2).

#### Dairy group

During the initial lifestyle discussion, 25 (48.1 %) women set 27 goals relating to dairy (Table 2). Of the goals set, 13 goals focused on improving diet quality by choosing low fat dairy products (Table 2). The remaining 14 goals were to adjust number of serves per day according to the dietary recommendations, including 13 goals to increase dairy intake and one goal to reduce dairy intake. Of these, the majority were set to meet the dietary recommendations (12, 85.7 %) and one (7.1 %) was more than the recommendation (Table 3).

After 1 month, the majority of women (*n* = 20, 80 %) successfully achieved 22 goals they set, which included increasing dairy intake (10 goals) and choosing low fat dairy food (12 goals). One (4.0 %) woman, who set one goal to reduce her daily dairy intake, reported reduced daily dairy intake but did not achieve her goal.

#### Meat and other protein-rich food group

During the initial lifestyle discussion, 25 (48.1 %) women set 26 goals relating to meat and other protein rich food (Table 2). Of the goals set, seven goals aimed to improve diet quality by choosing lean meat (6 goals) and including more red meat in diet (1 goal). The remaining 19 goals were to adjust the daily serves of protein food intake, including 14 goals aimed to reduce and five goals to increase meat intake. Of these, only four (21.1 %) met the recommendations, 10 (52.6 %) were more than the recommended daily serves, and three (15.8 %) were less than the recommendations.

After 1 month, most women (*n* = 15, 60.0 %) achieved their goals including reducing meat intake (six goals), increasing meat intake (five goals) and choosing lean meat (four goals). One (4.0 %) woman who set a goal to reduce her daily meat intake reported decreased meat intake after 1 month but did not completely achieve her goal. Two (8.0 %) women, who planned to reduce their daily meat intake, reported an increased intake after 1 month (Table 2).

#### Extra food group including foods high in sugar, fat and/or salt and those do not belong to any of the above mentioned five main food groups

This food group was the most frequently targeted by the women among the seven dietary goal categories. Thirty-five (67.3 %) women set a total of 40 goals during the initial lifestyle discussion (Table 2). The most frequently proposed goal was to limit food high in sugar, fat and/or salt (27 goals) followed by limiting fats and oil intake (eight goals) (Table 2). After 1 month, eight (22.9 %) women achieved eight goals they set included reducing fat, oil and/or sweets intake; two (5.7 %) women who set two goals reported they reduced their intake of soft drink and chocolate but had not fully achieved their goals; four (11.4 %) women who set four goals did not achieve their goals of decreasing chips, biscuits, ice-cream and cakes after 1 month (Table 2). For 23 (65.7 %) women who set a total of 26 goals, whether they achieved their goals is not known as there was insufficient information recorded in the booklets returned (Table 2).

#### Overall goals

Twenty-three (44.2 %) women set a total of 34 overall goals. Of these goals set, 20 aimed for maintaining their existing dietary intake, 13 were set to maintain their existing good dietary habits and one goal was set to reduce overall serve sizes across the five main food groups as the woman had a relatively balanced, but oversized, diet (Table 2).

After 1 month, 22 (95.7 %) women achieved 32 goals they set to either maintain their existing dietary intake or existing good dietary habits.

### Exercise goal and achievement

During the initial lifestyle discussion, 53 women proposed a total of 65 exercise goals from six different categories including to increase walking, maintain existing active lifestyle, increase recreational exercise, increase incidental exercise, adjust exercise intensity and improve overall fitness (Table 4).

**Table 4:** Exercise goals women set and whether achieved after 1 month

Exercise goal categories	Set goals	Achieved goals	Improved but did not achieved	Did not change	Worsened	Unknown
	*N* (%)	*N* (%)	*N* (%)	*N* (%)	*N* (%)	*N* (%)
Increase walking	28 (63.6)	7 (25.0)	6 (21.4)	8 (28.6)	2 (7.1)	5 (17.9)
Maintain exercise pattern	22 (50.0)	19 (86.4)	-	-	3 (13.6)	-
Increase recreational exercise	8 (18.2)	4 (50.0)	1 (12.5)	2 (25.0)	-	1 (12.5)
Increase incidental exercise	4 (9.1)	2 (50.0)	-	2 (50.0)	-	-
Adjust exercise intensity	2 (4.5)	1 (50.0)	-	-	-	1 (50.0)
Improve overall fitness	1 (2.3)	-	-	-	-	1 (100.0)

The most frequently proposed exercise goal by women was to increase walking (28 women, 28 goals). After 1 month, seven (25.0 %) women had achieved their goals, six (21.4 %) women had improved their walking exercise but had not achieved their goals, eight (28.6 %) women did not change their walking exercise, two (7.1 %) women reported a poorer exercise pattern where their walking exercise was decreased and for five (17.9 %) women it is not known if they had achieved their goals (Table 4).

A total of 22 (50.0 %) women set a goal of maintaining their existing active exercise pattern (Table 4). The majority of women (19 women, 86.4 %) achieved their goals successfully after 1 month but three (13.6 %) women reported a poorer exercise pattern during the review after 1 month where their physical activity levels were decreased.

During the initial lifestyle discussion, eight (18.2 %) women set a total of eight goals to increase their recreational exercise that included hydrotherapy, swimming, yoga, Pilates and pelvic floor exercises (Table 4). After 1 month, four (50.0 %) women achieved their goals; one (12.5 %) woman increased her recreational exercise but did not achieve the goal she set; one (12.5 %) woman did not change her recreational exercise pattern and for two women it is unknown if they achieved their recreational exercise goals (Table 4).

During the initial lifestyle discussion, four (9.1 %) women set four goals for increasing incidental exercise that included gardening and housework. After 1 month, two women (50 %) achieved their goals and the remaining two women (50 %) did not change their incidental exercise pattern (Table 4).

Two (4.5 %) women set two goals to adjust their exercise intensity during pregnancy, which included increased walking intensity and decreased gym exercise intensity. After 1 month, one (50 %) woman achieved her goal and the other 50 % woman did not report if she achieved her goal (Table 4).

## Discussion

We found that, after a month, women were likely to achieve goals for grains, fruits, dairy foods and overall diet, but less likely to meet goals targeting vegetables and limiting extra foods (including foods high in fat, sugar and/or salt and those not belonging to any of the five main food groups).

In our study, a high proportion of women reported their dietary intakes were lower than the recommendations for categories of grains, vegetables, fruits and dairy, therefore most goals were to increase daily intakes of food from these categories. While women’s daily intakes for meat and alternatives, and food from the extra food group category were more likely to be more than the recommendation, with most goals set to reduce daily intakes. Our findings were consist with the results from large observational studies, where Australian pregnant women reported lower consumption of all food groups, except for meat (and alternatives) and discretionary foods [27, 28]. Only a small proportion of women in our study seemed able to set and achieve a goal to meet the dietary recommendations by increasing their grain intakes or decreasing meat intakes.

For exercise goals, women who were already active and aimed to maintain their existing exercise pattern were more likely to be successful. Other exercise goals proposed by women, including increasing walking, increasing recreational exercise, adjusting exercise intensity and improving level of fitness all had a relatively low achievement rate after 1 month.

Few studies have reported on the use of a printed lifestyle tool to assist women with pregnancy hyperglycaemia in making changes to their dietary and exercise habits. Our findings help to provide in-depth understanding of the use by women of a printed lifestyle tool, their ability to assess their current diet and exercise patterns and their willingness to set lifestyle goals. This information may help to design behavioural intervention tools in the future and providing tailored care for women needing such advice.

A limitation of this study is that women were invited, but not required, to return booklets, and only a minority of women did so; in addition, the majority of women (94.6 %) who returned their booklets were from two geographical areas (Adelaide, South Australia and Melbourne, Victoria, Australia), which may limit generalisability of the study findings. In our study, information from the pregnancy record booklet about women’s diet and exercise changes was self-reported, so there may be differences between what women reported and what their diet and exercise patterns actually were. We were unable to assess whether goals achieved within the first month were sustained beyond this time. Longer term follow-up to assess the goals women set and whether able to be achieved over a longer period will be worth considering in any future studies.

## Conclusions

This descriptive study suggests that most dietary and exercise goals are achieved by women with borderline GDM who used a printed pregnancy record booklet. Women’s dietary goals targeting grains, fruits, dairy foods and overall dietary pattern are more likely to be achieved while dietary goals for improving vegetable and foods high in fat, sugar and/or salt and those do not belong to any of the five main food groups were less often achieved. Maintaining women’s existing active lifestyle is feasible during pregnancy although goals to increase physical activities are not as likely to be reached.

Providing a printed pregnancy record booklet may be beneficial in encouraging and assisting behavioural changes during pregnancy, although further assessment in different populations with longer term follow-up is warranted.

### Ethics approval and consent to participate

Human research ethics approval was obtained from the Children, Youth and Women's Health Service (CYWHS) Human Research Ethics Committee (REC 1860/8/12) for the IDEAL Study [23]. Written consent from participants was obtained. The lead investigator for the IDEAL Study, C A Crowther, provided permission for use of the IDEAL Study data for this manuscript.

### Consent for publication

Not applicable.

### Availability of data and materials

The datasets supporting the conclusions of this article are included within the article.

## Additional file

Additional file 1:
**Sample pages from the pregnancy record booklet.** Description of data: sample pages from the pregnancy record booklet for dietary review, dietary goal setting, exercise review and exercise goal setting. (PDF 1056 kb)

## Abbreviations

ARCH: Australian Research Centre for Health of Women and Babies
BMI: Body mass index
GDM: Gestational diabetes mellitus
GI: Glycaemic index
IDEAL Study: Investigation of dietary advice and lifestyle for women with borderline gestational diabetes
MNT: Medical nutrition therapy
OGCT: Oral glucose challenge test
OGTT: Oral glucose tolerance test
