# Supplementary material for: Assessing use of a printed lifestyle intervention tool by women with borderline gestational diabetes and their achievement of diet and exercise goals: a descriptive study
Source: BMC Pregnancy Childbirth. 2016 Mar 1;16:44. doi: 10.1186/s12884-016-0825-z (PMC4774025; doi:10.1186/s12884-016-0825-z)
Supplement: Additional file 1: — Sample pages from the pregnancy record booklet. Description of data: sample pages from the pregnancy record booklet for dietary review, dietary goal setting, exercise review and exercise goal setting. (PDF 1056 kb) [file 12884_2016_825_MOESM1_ESM.pdf]

Today's date: _ _ / _ _ / _ _

Visit 1: Dietary Review

How many serves of each food group do you eat on an average **day**?

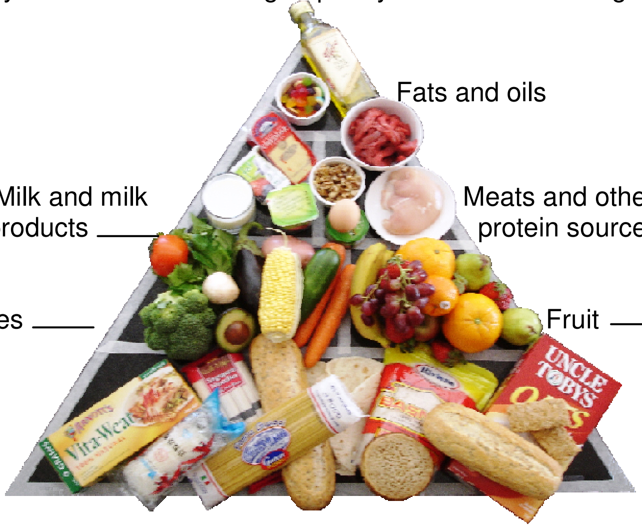

Fats and oils

Milk and milk products _____

Meats and other protein sources _____

Vegetables _____

Fruit _____

Breads, cereal, grains, rice, pasta _____

Good things I already do:

- ☐ choose wholemeal
- ☐ choose low fat
- ☐ choose unsaturated oils
- ☐ choose low GI foods
- ☐ others: ____________________
- ☐ choose wholegrain
- ☐ choose lean meat
- ☐ limit fats and oils
- ☐ limit tropical fruits

Healthy, low GI choices and good choices I have been making in each food group:

- Breads, cereals, grains, rice, pasta
- Vegetables
- Fruit
- Meat and other protein sources
- Milk and milk products
- Fats and oils

Today's date: _ _ / _ _ / _ _

My Dietary Goals

Refer to your information booklet to help with completing this page.

Write down any changes to the number of serves of each food group you will aim for each day.

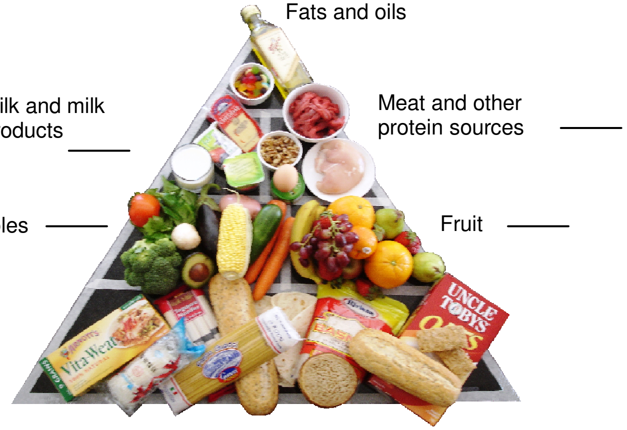

Fats and oils

Milk and milk products _____

Meat and other protein sources _____

Vegetables _____

Fruit _____

Breads, cereals, grains, rice and pasta _____

Good and low GI choices I will make this month in each food group

- Breads, cereals, grains, rice, pasta
- Vegetables
- Fruit
- Milk and milk products
- Meat and other protein sources
- Fats and oils

Foods I will avoid this month

QUESTIONS??____________________

Remember to discuss this with your doctor or midwife at your antenatal visit.

Today's date: _ _ / _ _ / _ _

Visit 1: Exercise Review

Incidental exercise

List the things you do as part of your everyday life that provide some exercise:
(eg cooking, ironing, gardening, walking to work)

____
____
____

How much time would you normally spend on these tasks?

__________ hrs/day

OR __________ hrs/week

Recreational exercises

Fill in as many of these as required:

Type of exercise: __________<br>How many times a week? ________<br>Intensity (tick one or more):<br>☐ light<br>☐ moderate<br>☐ intense<br>How long is each session? ______	Type of exercise: __________<br>How many times a week? ________<br>Intensity (tick one or more):<br>☐ light<br>☐ moderate<br>☐ intense<br>How long is each session? ______
Type of exercise: __________<br>How many times a week? ________<br>Intensity (tick one or more):<br>☐ light<br>☐ moderate<br>☐ intense<br>How long is each session? ______	Type of exercise: __________<br>How many times a week? ________<br>Intensity (tick one or more):<br>☐ light<br>☐ moderate<br>☐ intense<br>How long is each session? ______

Today's date: _ _ / _ _ / _ _

My Exercise Goals

Have you spoken about exercise with your:

☐ Midwife
☐ Obstetrician/GP
☐ Partner
☐ Other __________

Fill in a few goals for your exercise regime over the next month

What I will try in between now and my next visit:

- Changes to current activities:
  -
  -
  -
- Changes to timing and frequency:
  - ☐ Adding 5 mins to every session
  - ☐ Adding an extra day during the week
  - ☐ Other: __________
- New activities:
  -
  -

My other exercise goals:

-
-
-

QUESTIONS??______________________________
____

Remember to discuss this with your doctor or midwife at your antenatal visit..
